# Short-term warfarin treatment for apical thrombus in a patient with Takotsubo cardiomyopathy

**DOI:** 10.5830/CVJA-2016-011

**Published:** 2016

**Authors:** Abdullah İcli, Hakan Akilli, Mehmet Kayrak, Alpay Aribas, Kurtulus Ozdemir

**Affiliations:** Department of Cardiology, Meram School of Medicine, Necmettin Erbakan University, Konya, Turkey; Department of Cardiology, Meram School of Medicine, Necmettin Erbakan University, Konya, Turkey; Department of Cardiology, Meram School of Medicine, Necmettin Erbakan University, Konya, Turkey; Department of Cardiology, Meram School of Medicine, Necmettin Erbakan University, Konya, Turkey; Department of Cardiology, Meram School of Medicine, Necmettin Erbakan University, Konya, Turkey

**Keywords:** Takotsubo cardiomyopathy, apical thrombus, warfarin

## Abstract

Takotsubo cardiomyopathy (TCMP) is characterised by a temporary aneurysm of the left ventricular apex in individuals without significant stenosis of the coronary arteries. It is extremely rare to see it combined with a thrombus. In this case report, we present a 57-year-old female patient with TCMP in whom apical thrombus was treated with short-term warfarin use.

## Abstract

Takotsubo cardiomyopathy (TCMP) is characterised by a temporary aneurysm of the left ventricular apex in individuals without significant stenosis of the coronary arteries. Mostly seen in postmenopausal women, it is also called ampulla cardiomyopathy, human stress cardiomyopathy or broken heart syndrome.^1^ It is extremely rare to see it combined with a thrombus.

The Mayo Clinic diagnostic criteria for TCMP include reversible left ventricular dysfunction, newly emerging ECG changes and/or increased troponin levels, intracranial haemorrhage, pheochromocytoma and hypertrophic cardiomyopathy, absence of head trauma, and angiographic exclusion of occlusive coronary artery disease or plaque rupture.^2^ In this case report, we present a 57-year-old female patient with TCMP in whom apical thrombus was treated with short-term warfarin use.

## Case report

A 57-year-old postmenopausal female patient was admitted to the emergency department with a four-day history of chest pain and dyspnoea. Her past medical history included hypertension.

**Fig. 1:**
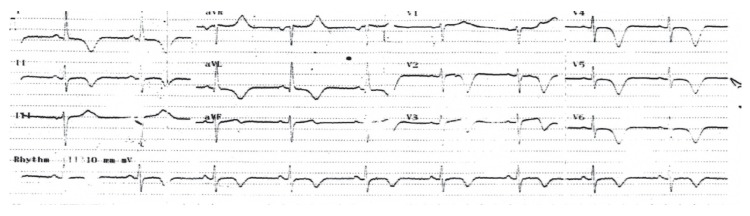
Admission ECG showing ST–T changes.

**Fig. 2:**
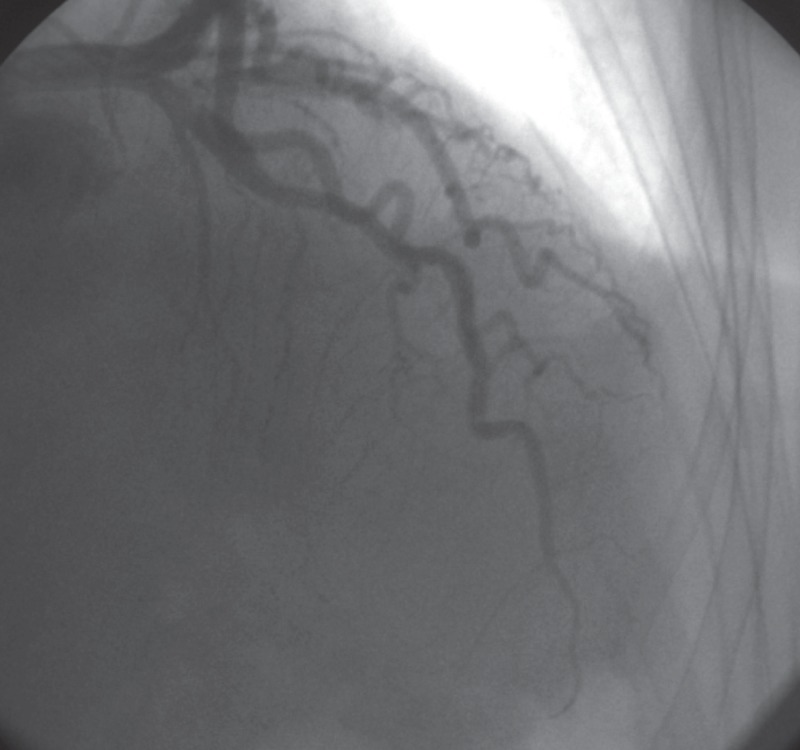
Coronary angiography with normal coronary angiographic findings.

**Fig. 3:**
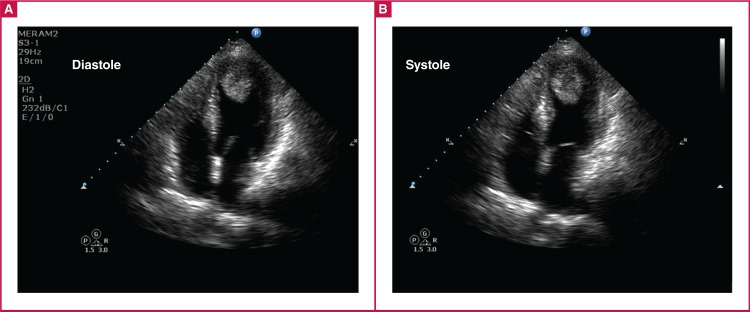
Initial echocardiography showing apical ballooning and apical thrombus in the diastolic and systolic phase.

Electrocardiography performed in the emergency department showed symmetrical T-wave negativity in V1–V6 and DI–avL (Fig. 1). With ongoing chest pain, the patient underwent coronary angiography, which detected normal coronary anatomy (Fig. 2). During the follow up, the troponin level was 0.83 ng/ ml. Transthoracic echocardiography revealed a dyskinetic left ventricular apex, with an ejection fraction of 35% and a 2.3 × 3.3-cm thrombus (Fig. 3).

In the light of the typical ECG, coronary angiography and echocardiography findings, the patient was diagnosed with TCMP. The patient was informed about the risks and benefits of anticoagulation with warfarin, surgical thrombectomy and other treatment options, including beta-blockers and angiotensin converting enzyme inhibitor. Warfarin was commenced. The patient was discharged with a recommendation to visit a week later for measurement of the prothrombin time international normalised ratio (PT-INR) and warfarin dose arrangement.

**Fig. 4:**
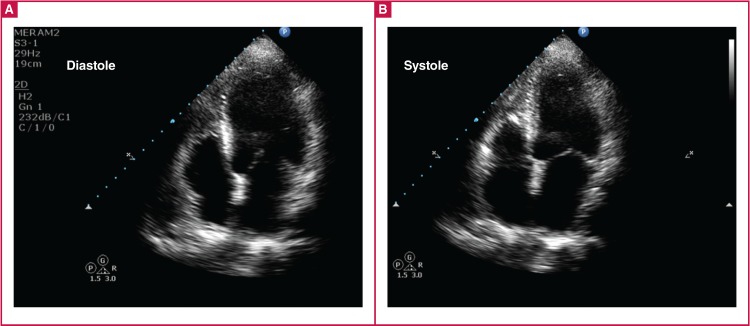
After warfarin treatment, echocardiography showing improved left ventricle and resolved thrombus in the diastolic and systolic phase.

Fifteen days later, the patient was admitted with bruising on her body, and her PT-INR level was 6.5. The echocardiographic examination was repeated, which showed that the apical dyskinesia and thrombus in the left ventricle had disappeared, and the ejection fraction was normal (Fig. 4).

## Discussion

The vast majority (90%) of patients with TCMP are hypertensive postmenopausal women.^3^ In addition to ST-segment elevation, other ECG changes such as T-wave inversion and QT prolongation may be seen. Cardiac enzymes are generally moderately elevated. For these reasons, TCMP is often misdiagnosed as myocardial infarction with ST elevation.

A definitive diagnosis is made with the detection of hypokinetic and aneurysmal images of the left ventricular apex in echocardiography or ventriculography, with coronary angiography showing an absence of stenosis in the coronary arteries.^4^ Cardiac magnetic resonance imaging may be highly beneficial in differentiating between various types of cardiomyopathy and myocarditis.^5^

Diverse factors have been proposed for the pathophysiology of TCMP, including stress, increased adrenergic activity, prolonged stunned myocardium, hypertension, chronic obstructive lung disease, decreased oestrogen levels, small-vessel disease, myocarditis and insufficient fatty acid metabolism in the myocardium.^6^ The mortality rate from TCMP is lower than that of acute myocardial infarction. In-hospital mortality is quite low, at 1–2%.^1^,^4^ The complications include apical thrombus formation, cardiac rupture, embolism and conduction defects.^7^

De Gregorio *et al.* (2008) reported intracavitary thrombus in 2.5% of the patients with TCMP, and stated that 33% of these patients may have thromboembolic complications.^8^ However, thromboembolic events may occur even in patients receiving anticoagulant treatment.^7^ Myocardial necrosis and haemorrhage are feared limitations in treatment decisions.^9^ Surgical thrombectomy has drawbacks, such as decreasing the ejection fraction in the early post-surgical period, and the increased risks of anaesthesia and operational stress for patients with TCMP.^10^,^11^

## Conclusion

This patient’s outcome shows that anticoagulant treatment with warfarin is an effective, conservative treatment option. Despite ongoing debate, it would be beneficial to consider warfarin in individualised treatment, and the decision should be made with consideration of the features of intracavitary thrombus.
